# Sex differences in opioid response: a role for the gut microbiome?

**DOI:** 10.3389/fphar.2024.1455416

**Published:** 2024-08-29

**Authors:** Caitlin Han, Melissa T. Manners, Shivon A. Robinson

**Affiliations:** ^1^ Department of Psychology, Williams College, Williamstown, MA, United States; ^2^ Department of Biological and Biomedical Sciences, Rowan University, Glassboro, NJ, United States

**Keywords:** opioids, microbiome, sex differences, estrogen, behavior

## Abstract

Opioid drugs have been long known to induce different responses in males compared to females, however, the molecular mechanisms underlying these effects are yet to be fully characterized. Recent studies have established a link between the gut microbiome and behavioral responses to opioids. Chronic opioid use is associated with gut dysbiosis, or microbiome disruptions, which is thought to contribute to altered opioid analgesia and reward processing. Gut microbiome composition and functioning have also been demonstrated to be influenced by sex hormones. Despite this, there is currently very little work investigating whether sex differences in the gut microbiome mediate sex-dependent responses to opioids, highlighting a critical gap in the literature. Here, we briefly review the supporting evidence implicating a potential role for the gut microbiome in regulating sexually dimorphic opioid response and identify areas for future research.

## Introduction

The opioid epidemic is a growing public health crisis in the United States. In 2022, over 80,000 people died from an opioid-related overdose (CDC, 2024). Opioids are powerful painkillers, and while they pose the risk of potential negative side effects, such as dependence, hyperalgesia, and respiratory depression (Higgins et al., 2019; Algera et al., 2021), many people rely on these drugs as a form of pain management. Over 80% of patients who undergo low-risk surgery receive opioids for postoperative pain management and more than 60% of patients who receive postoperative opioid therapy become chronic opioid users following treatment (Sun et al., 2016; Hah et al., 2017). Furthermore, an estimated 5–8 million Americans with chronic pain use opioids for long-term care (Reuben et al., 2015). On average, 8%–12% of patients using opioids to treat chronic pain develop opioid use disorder (OUD), which is characterized by uncontrollable cravings and hazardous patterns of drug use that interfere with daily functioning (Vowles et al., 2015; Pergolizzi et al., 2020).

Sex differences have been demonstrated in opioid-induced antinociception, reward, tolerance, withdrawal, and hyperalgesia (Bodnar and Kest, 2010). While the neurobiological processes underlying sex-specific opioid effects are yet to be fully characterized, some proposed mechanisms include differences in opioid metabolism, ligand-receptor dynamics, endocrine signaling, and neuroimmune activation, in addition to sex-gene interactions (Bodnar and Kest, 2010; Fullerton et al., 2018; Averitt et al., 2019). A much less explored variable is the gut microbiome, a network of bacteria and other microorganisms that colonize the gastrointestinal (GI) tract. Despite their localization in the GI tract, gut microbiota can have widespread effects on the functioning of various systems within the body. Of great relevance for opioid signaling is the gut-brain axis, which mediates bidirectional communication between the gut and the central nervous system in part through gut-derived neuroactive metabolites and neurotransmitters (Cryan et al., 2019). For example, short-chain fatty acids (SCFAs), the main metabolite produced by microbes in the gut, are implicated in brain processes that contribute to neuroimmune functioning, stress response, and emotional affect (Silva et al., 2020). A growing body of literature implicates the role of sex hormones in modulating microbiome composition and function (Yoon and Kim, 2021). Sex differences in the gut microbiome have also been described in several gastrointestinal (Kim, 2022), autoimmune (Rizzetto et al., 2018), cardiovascular (Razavi et al., 2019), and neuropsychiatric disorders (Shobeiri et al., 2022).

Opioids produce their analgesic and mood-altering effects via interactions with the endogenous opioid system, which includes mu (MOR), kappa (KOR), and delta receptors (DOR) and their respective neuropeptide ligands, which are widely expressed throughout the central and peripheral nervous systems (Schepper et al., 2004; Emery and Akil, 2020). Opioid receptors are also present in the enteric nervous system, a division of the peripheral nervous system composed of sensory and motor neurons that regulate gut functioning (Wood and Galligan, 2004). Chronic opioid use impairs gastric motility, intestinal permeability, and bowel function, which in turn can disrupt gut microbiome homeostasis (Kurz and Sessler, 2003; Wang and Roy, 2017; Rueda-Ruzafa et al., 2020). Opioid-indued gut dysbiosis, or dysregulation of the gut microbiome, is thought to mediate changes in behavioral response to opioids over time and may contribute to the development of substance use disorders (Meckel and Kiraly, 2019; Angoa-Pérez and Kuhn, 2021). However, whether sex differences in the microbiome impact these processes is unclear. Elucidating the neurobehavioral effects of opioids in the context of the gut microbiome, and how these mechanisms may be differentially affected by sex, is important for predicting opioid response and identifying risk factors. In this review, we discuss recent findings from the literature investigating sex differences in opioid response, sex differences in the gut microbiome, and the role of the microbiome in opioid response to highlight the need for further research to understand the relationship between sex-specific gut microbiome activity and sex differences in opioid response.

## Sex differences in response to opioids

Opioids are well-known to induce divergent effects in males versus females. Men are diagnosed with OUD at a higher rate (Wilson et al., 2020) and are more likely to overdose on opioids compared to women (Butelman et al., 2023), whereas women report less pain relief following opioid treatment (Cepeda and Carr, 2003; Aubrun et al., 2005; LeResche et al., 2015) and experience more pronounced adverse side effects, such as nausea and vomiting (Fillingim et al., 2005). In rodent models, males generally exhibit more potent antinociceptive responses to opioids compared to females (Craft, 2003) while females appear to be more sensitive to the reinforcing effects of opioids (Craft, 2008; Karami and Zarrindast, 2008; Fulenwider et al., 2020; Phillips et al., 2020). However, it is important to note that these observations may depend on the specific opioid, dose, and duration of treatment implemented.

The natural fluctuation of gonadal hormones during the female reproductive cycle likely contributes to differences in opioid response in males versus females (Fillingim and Ness, 2000). The human reproductive cycle, known as the menstrual cycle, lasts for 28 days on average (Reed and Carr, 2015) and is commonly divided into the menstrual, follicular, ovulatory, luteal, and pre-menstrual phases (Joyce et al., 2021). Estrogen levels increase during the follicular phase and peak prior to ovulation while progesterone levels peak during the luteal phase. During menopause, ovarian estrogen production decreases significantly (Al-Azzawi and Palacios, 2009). Very few studies have directly investigated opioid response in pre-menopausal versus post-menopausal women, however, sex differences in opioid analgesia are notably diminished in elderly patients (Aubrun et al., 2005; LeResche et al., 2015).

The murine estrous cycle is analogous to the human menstrual cycle and begins 4–6 weeks after birth (Ajayi and Akhigbe, 2020). The cycle consists of four phases–proestrus, estrus, metestrus, and diestrus–and lasts approximately 4–5 days. The proestrus phase corresponds with elevated estrogen levels, whereas the metestrus and diestrus phases are associated with elevated progesterone levels (Goldman et al., 2007; Ajayi and Akhigbe, 2020).

In normally cycling female rats, morphine’s analgesic effects are most potent during the proestrus phase (Stoffel et al., 2003; Terner et al., 2005). In contrast, heroin self-administration is lowest during the proestrus phase (Lacy et al., 2016). However, some studies have found periods of elevated estradiol levels to be associated with enhanced MOR-dependent synaptic plasticity (Harte-Hargrove et al., 2015) and increased demand for remifentanil self-administration using a threshold procedure (Lacy et al., 2020). Daily treatment with estradiol increases heroin self-administration in ovariectomized animals (Roth et al., 2002), though, this effect appears to be dose-dependent since higher doses of estradiol reduce heroin self-administration in both intact (Sharp et al., 2021) and ovariectomized females (Smith et al., 2021). When tested for morphine conditioned place preference (CPP), ovariectomized females exhibit less drug-seeking behavior compared to intact animals (Mirbaha et al., 2009).

Gonadectomy experiments have also revealed a causal effect of sex hormones on pain processing. Gonad removal decreases opioid analgesia in males and increases analgesia in females (Terner et al., 2002; Stoffel et al., 2003), suggesting that testosterone promotes opioid antinociception whereas estradiol has the opposite effect (Xu et al., 2024). Indeed, estradiol treatment in ovariectomized females reduces the effectiveness of opioids in attenuating pain (Ji et al., 2007). Furthermore, estradiol upregulates the expression of UDP-glucuronosyltransferase 1A9 (UGT1A9) (Cho et al., 2016), one of several enzymes within the UGT family that metabolizes opioids (Gregori et al., 2012). Following morphine exposure, females exhibit higher plasma levels of morphine-3-glucuronide (M3G), a metabolite that opposes the analgesic effects of morphine (Baker and Ratka, 2002; Doyle and Murphy, 2018). Altogether, these findings provide strong support for the modulation of opioid signaling by sex hormones.

Further research is needed to elucidate the relationship between the menstrual cycle and opioid response in humans. Some studies indicate that women experience greater opioid analgesia during the follicular phase compared to the luteal phase (Ribeiro-Dasilva et al., 2011; Miniksar et al., 2023), whereas others report no differences in opioid pain relief across the menstrual cycle (Sener et al., 2005; Ahmed et al., 2012). While there is evidence suggesting that ovarian hormones influence addictive behaviors in the context of alcohol and nicotine use (Joyce et al., 2021; Towers et al., 2022), there is a striking lack of studies investigating interactions between the menstrual cycle and opioid use.

## Sex differences in the gut microbiome

The human gut contains over 10^14^ bacteria which are primarily represented by six phyla: *Firmicutes*, *Bacteroidetes*, *Actinobacteria*, *Proteobacteria*, *Fusobacteria*, and *Verrucomicrobia* (Arumugam et al., 2011). Among the diverse range of bacterial species that inhabit the gut, some are considered “good” bacteria, or health-promoting, while others are pathogenic (Zaky et al., 2021), For example, *Lactobacillus* and *Bifidobacterium* bacterial strains are generally considered to be beneficial (Reid, 1999; Cronin et al., 2011), and are used in treating several gut and bowel-related diseases (O’Callaghan and Sinderen, 2016; Li et al., 2023). In contrast, alterations in the ratio of abundance of *Firmicutes* to *Bacteroidetes* are associated with obesity and inflammatory bowel disease (Stojanov et al., 2020).

The colonization of the gut begins immediately following birth via exposure to maternal vaginal and fecal microbiota along with microbes introduced from the environment (Vaishampayan et al., 2010). Although there are some heritable components of the gut microbiome (Hall et al., 2017), nongenetic factors such as birth method (i.e., vaginal versus caesarean section), diet (i.e., formula vs. breastmilk), medication, and other early life experiences can modulate the development of the gut microbiome (Turroni et al., 2014; Knoop et al., 2018; Hantsoo and Zemel, 2021). The gut microbiome typically stabilizes during toddlerhood; however, it can continue to be influenced by various environmental and psychosocial factors throughout the lifespan (Yatsunenko et al., 2012; Badal et al., 2020).

The composition of the gut microbiome varies depending on sex. A sex-specific examination of the gut microbiota of 1,135 individuals demonstrated that women have increased microbial diversity compared to men (Sinha et al., 2019). The relative abundance of SCFA-producing bacterial genera, such as *Prevotella,* is higher in pre-menopausal women compared to men, while post-menopausal women have a similar level of abundance to men (Santos-Marcos et al., 2018). The analysis of 89 different inbred mice strains revealed that the commonly used C57BL/6J strain has one of the most prominent sexually dimorphic microbiomes, with females harboring a higher abundance of *Coprococcus* and *Bacteroides* compared to males (Org et al., 2016).

Sex differences in the microbiome are mediated in part by gonadal hormones. Men and women with high serum levels of testosterone and estradiol, respectively, exhibit greater diversity in gut microbiota compared to those with low concentrations (Shin et al., 2019). In rodent models, gonadal hormones produced during puberty make the male gut microbiome less diverse than females (Yurkovetskiy et al., 2013). Notably, male mice castrated before puberty show a similar level of species diversity to that of intact females. Testosterone administration also has a protective effect against the abnormal gut colonization observed in gonadectomized pathogen-free male mice who were otherwise untreated, indicating a role of sex hormones in determining the composition of the gut microbiome (Org et al., 2016). Furthermore, genetic deletion of estrogen receptor β (ERβ), the primary estrogen receptor in the colon (Langen et al., 2011), significantly alters gut microbiome composition in mice (Menon et al., 2013; Ma et al., 2022).

The gut microbiome plays a reciprocal role in modulating the production of sex hormones (Baker et al., 2017). The gut microbe *Clostridium scindens* has been shown to convert glucocorticoids into androgens in humans (Ridlon et al., 2013). Several different gut bacteria, including the genera *Clostridium, Bacteroides, and Bifidobacterium*, produce the enzyme β-glucuronidase (βGLU), which metabolizes endogenous and exogenous compounds including drugs, neurotransmitters, and hormones (Kwa et al., 2016; Gao et al., 2022). Specifically, βGLU is important for converting estrogen into its active form at which point it can induce its physiological effects (Hu et al., 2023). Post-menopausal women exhibit alterations in the diversity of βGLU producing bacteria (Flores et al., 2012; Silva et al., 2022; Hu et al., 2023), although an increased abundance of the *Ruminococcus* genus is positively correlated with estrogen metabolites among this population (Fuhrman et al., 2014; Wu et al., 2023).

## Opioid use affects the gut microbiome

Chronic opioid use disrupts gut homeostasis in humans (Acharya et al., 2017; Barengolts et al., 2018; Gicquelais et al., 2020; Cruz-Lebrón et al., 2021; Yan et al., 2023). However, human studies are often limited by small sample sizes, polydrug use, and differences in dietary patterns, thus making it difficult to draw mechanistic conclusions. Animal studies have been crucial in identifying links between opioid-induced microbiome disruptions and changes in drug response (Akbarali and Dewey, 2017; Meckel and Kiraly, 2019). Several studies report the depletion of the *Lactobacillus* and *Bifidobacterium* bacterial strains in opioid-exposed rodents (Zhang et al., 2019; Abu et al., 2024; Muchhala et al., 2024). Opioid exposure is also associated with alterations in the abundance of *Firmicutes* and *Bacteroidetes* in the gut (Banerjee et al., 2016; Simpson et al., 2020; Zhang et al., 2021; Antoine et al., 2022; Blakeley-Ruiz et al., 2022; Ghosh et al., 2023; Truitt et al., 2023; Crawford et al., 2024; Muchhala et al., 2024). Opioid-induced gut dysbiosis is also reported in gestational and neonatal exposure models (Grecco et al., 2021; Abu et al., 2022; 2024; Antoine et al., 2022; Lyu et al., 2022), which may have important implications for neonatal opioid withdrawal syndrome symptoms (Sealschott et al., 2020; 2024). Lastly, the mechanism of opioid administration can also affect the composition of the gut microbiome. Intermittent opioid intake via injections is more disruptive to gut homeostasis than sustained administration through a slow-release subcutaneous pellet (Lee et al., 2018), likely due to the recurring periods of withdrawal that are associated with intermittent exposure. Furthermore, intermittent opioid treatment is more likely to induce neuroimmune activation, hyperalgesia, and enhanced dopamine signaling in the nucleus accumbens (Lee et al., 2018; Lefevre et al., 2020).

Opioid-exposed animals supplemented with SCFAs exhibit reduced analgesic tolerance (Muchhala et al., 2024) and conditioned place preference (Hofford et al., 2021) compared to non-treated animals. SCFAs have also been shown to mediate the reversal of opioid-induced hyperalgesia (Jessup et al., 2023; Crawford et al., 2024). Microbiome depletion via antibiotic treatment attenuates withdrawal severity (Thomaz et al., 2021; Truitt et al., 2023) and reduces neuronal activation in brain regions implicated in opioid withdrawal (Simpson et al., 2020), suggesting that gut microbiota mediate the development of dependence. Antibiotic treatment has also been shown to attenuate opioid tolerance (Kang et al., 2017; Zhang et al., 2019) and drug-seeking behavior in the CPP (Hofford et al., 2021). However, in a separate study, antibiotic treatment was found to increase fentanyl intake during intravenous self-administration (IVSA), and this was eliminated by SCFA supplementation (Hofford et al., 2024). Thus, the relationship between the gut microbiome and opioid-induced behavior is complex, where increased or decreased microbial diversity could be beneficial depending on the circumstances.

## Conclusions and future implications

There is compelling evidence indicating that both sex hormones and opioids modulate the composition and activity of the gut microbiome. Importantly, opioids suppress the hypothalamic-pituitary-gonadal axis, which can lead to dysregulated endocrine functioning (Brennan, 2013; Marudhai et al., 2020; Wehbeh and Dobs, 2020). Chronic opioid use reduces the synthesis of testosterone in men (Rubinstein et al., 2013; Bawor et al., 2015) and estradiol and progesterone in women (Daniell, 2008). Furthermore, women who use opioids long-term are more likely to experience menstrual cycle abnormalities compared to short-term users (Daniell, 2008; Richardson et al., 2018). Thus, the effects of chronic opioid exposure on sex hormone regulation could in turn influence microbiome functioning in a sex-dependent manner.

Unfortunately, the majority of preclinical studies examining the effects of opioids on gut microbiota are conducted only in male animals or are not statistically powered to investigate sex differences. Despite this, notable sex differences have arisen among the small sample of studies that have examined the interaction between sex and gut microbiota in the context of opioid exposure (Table 1). Early-life opioid exposure differentially alters the gut microbiome in males versus females when measured later in life (Antoine et al., 2022; Lyu et al., 2022). In adults, IVSA induces distinct gut microbiome alterations in males compared to females, though no differences in addictive-like behavior were observed (Ren and Lotfipour, 2022b; Greenberg et al., 2024). In a separate study, microbiome depletion enhanced motivation for fentanyl at low schedules of reinforcement in males, but not females (Ren and Lotfipour, 2022a). Some studies, while including both males and females, did not explicitly test for sex differences in gut microbiome composition (Simpson et al., 2020; Grecco et al., 2021; Ghosh et al., 2023; Truitt et al., 2023). Thus, further investigation into the role of sex in opioid-induced gut dysbiosis and associated neurobehavioral effects is highly warranted. Future research should prioritize testing both male and female subjects and be designed to robustly evaluate sex as a biological variable (Radke et al., 2021; Shansky and Murphy, 2021). This is not to imply that the gut microbiome is the only factor driving sex differences in opioid response. However, this area of research is a promising avenue for gaining greater insight into the mechanisms governing opioid response, in addition to improving health outcomes for people who use opioids.

**TABLE 1:** Opioid-induced microbiome alterations in studies using males and females. IVSA = intravenous self-administration.

Reference	Species	Drug	Method of drug exposure	Age during drug exposure	Opioid-induced bacterial phyla or genus alteration	Behavioral/physiological alteration
Ren and Loftipour (2022b)	Sprague Dawley rats	Fentanyl	IVSA	Adult	↓*Verrucomicrobia* in males ↓*Akkermansia* in males ↑*Prevotella* in females ↑*Ruminococcus* in males	Gut diversity within females was correlated with progressive ratio schedules during fentanyl IVSA
Greenberg et al. (2024)	NIH heterogeneous stock rats	Heroin	IVSA	Adult	↑*Verrucomicrobia* in females ↑*Actinobacteria* in females ↑*Spirochaetes* in males ↑*Tenericutes* in males	Males and females did not differ in drug intake during heroin IVSA
Lyu et al. (2022)	CF1 mice	Oxycodone	Intraperitoneal injection (dams)	Gestational	↑*Saccharibacteria* in females ↑*Enterococcus* spp. in females ↑*Clostridia* in males and females ↑*Butyricimonas* spp. in females ↑*Bacteroidetes* in females ↑*Anaeroplasma* spp. in females ↑*Coriobacteriaceae* in males ↑*Roseburia* spp. in males ↑*Sutterella* spp. in males	Males and females did not differ in behavioral outcomes
Antoine et al. (2022)	C57BL/6 mice	Morphine	Subcutaneous injection	Neonatal	↓*Verrucomicrobia* in females ↓*Bacteroidetes* in females ↓*Actinobacteria* in females ↑*Firmicutes* in females	
Ren and Loftipour (2022a)	Sprague Dawley rats	Fentanyl	IVSA	Adult	*↑Bacteroidetes* in antibiotic-treated males Females not analyzed	Antibiotic treatment enhanced fentanyl self-administration in males, but not females, at the lowest fixed ratio schedule
Ghosh et al. (2023)	C57BL/6 mice	Morphine	Subcutaneous pellet	Adult	↓*Tenericutes* ↓*Actinobacteria* ↓*Bacteroidetes* ↑*Verrucomicrobia* ↑*Firmicutes* Sex differences not analyzed	
Truitt et al. (2023)	C57BL/6 mice	Morphine	Subcutaneous pellet or intraperitoneal injection	Adult	↓*Actinobacteria* ↓*Firmicutes* ↑*Verrucomicrobia* Sex differences not analyzed	Morphine-treated males and females did not differ in somatic withdrawal symptoms. Microbiome depletion prevented development of morphine withdrawal symptoms
Grecco et al. (2021)	C57BL/6 mice	Oxycodone/Methadone	Subcutaneous injections (dams)	Gestational	↑*Saccharimonadaceae* ↑*Peptococcaceae* ↑*Rikenellaceae* Sex differences not analyzed	
Abu et al. (2022)	C57BL/6 mice	Hydromorphone	Intraperitoneal injection (dams)	Gestational	*↑Firmicutes ↑Bacteoidetes ↑Actinobacteria* Sex differences not analyzed	

As opioids are commonly prescribed for postoperative pain (Hah et al., 2017), preventive interventions could be vital in managing adverse responses to opioids, particularly in high-risk populations. Dietary interventions targeted toward the gut microbiome could potentially attenuate the negative side effects of chronic opioid use. In mice undergoing oxycodone IVSA, treatment with dietary omega-3 polyunsaturated fatty acids diminished drug-seeking behavior during extinction and reinstatement and reduced anxiety-like behavior (Hakimian et al., 2019). Probiotics are commonly used for treating and preventing gut-related disorders (Liu et al., 2018), and more recently have gained traction as beneficial supplements to medications for certain psychiatric disorders (Forth et al., 2023). The utilization of adjuvant probiotic treatment with prescription opioids may reduce symptoms driven by gut dysbiosis. Indeed, a small randomized, double-blinded, placebo-controlled study found that probiotic supplementation reduced depression severity and metabolic dysfunction in patients undergoing methadone maintenance treatment (Molavi et al., 2022). However, additional studies with larger cohorts are needed to verify these findings.

Distinct gut microbial profiles have recently been identified in patients with Alzheimer’s disease (Ances et al., 2023), food addiction (Dong et al., 2020), and epilepsy (Gong et al., 2020), highlighting a growing interest in utilizing gut microbiome biomarkers as a diagnostic tool for a range of conditions. Moreover, sex-specific gut microbiome biomarkers have been discovered for major depressive disorder (Chen et al., 2018). Further characterization of sex-specific opioid-induced gut microbiome alterations could lead to implementing personalized medicine approaches based on microbial predictors (Ghanbari and Sumner, 2018). Thus, increased consideration of sex differences in the microbiome and its relationship to opioid response has significant therapeutic implications.
